# Supplementary figures and images for: Mucosal immunization with the lung Lactobacillus-derived amphiphilic exopolysaccharide adjuvanted recombinant vaccine improved protection against P. aeruginosa infection
Source: PLoS Pathog. 2024 Nov 18;20(11):e1012696. doi: 10.1371/journal.ppat.1012696 (PMC11611261; doi:10.1371/journal.ppat.1012696)

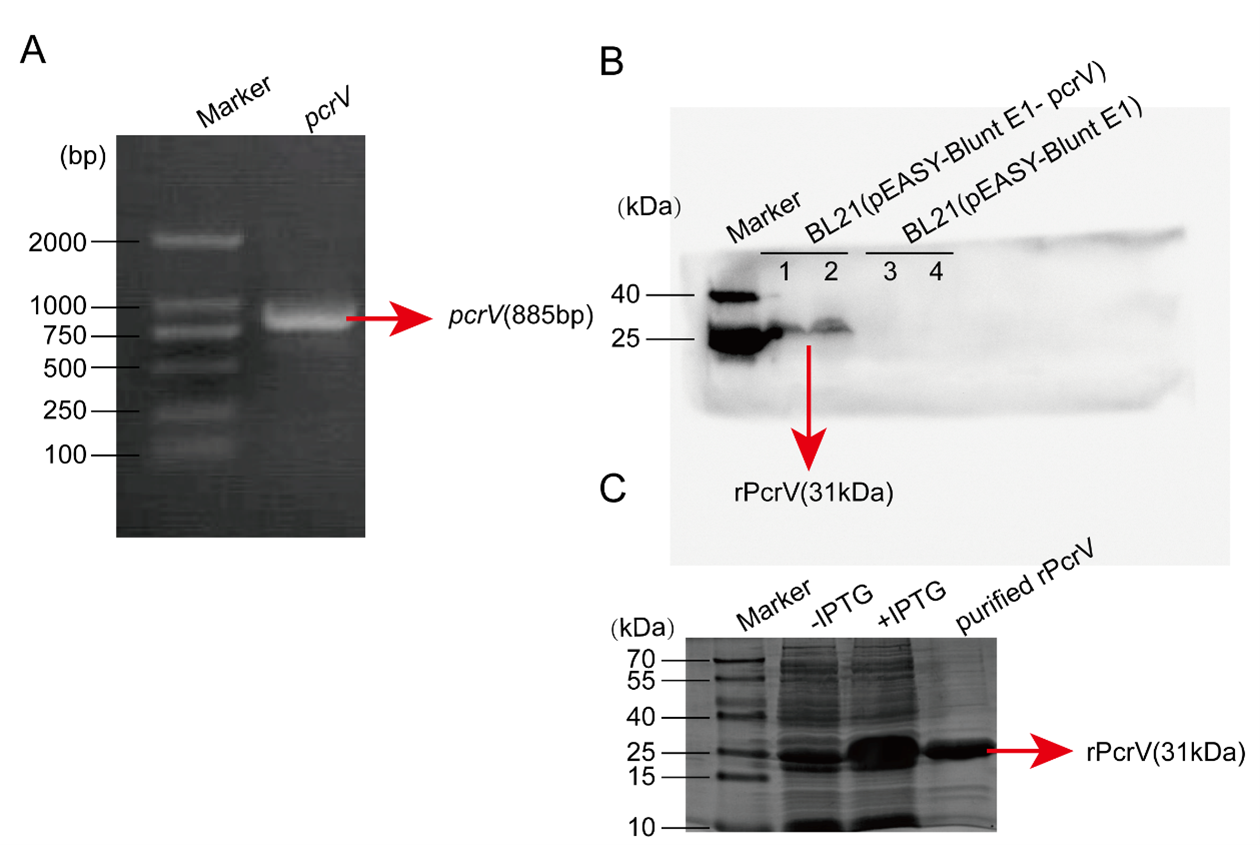

Supplement: S1 Fig — (A) pcrV gene sequence (885 bp) was was cloned from P. aeruginosa PAO1. Arrows: pcrV gene. (B) rPcrV expression was evaluated in vitro by Western blotting analysis. Lane M, standard size marker (kDa); lane1 and 2 cell lysis protein of BL21(pEASY-Blunt E1- pcrV); lane3 and 4 cell lysis protein of BL21(pEASY-Blunt E1). Arrows: rPcrV protein. (C) Samples were resuspended in SDS loading buffer and boiled for 5 min. Lane M, standard size marker (kDa); lane 1, pellet of non-induced bacteria; lane 2, pellet of IPTG-induced bacteria; lane 3, purified rPcrV from Ni-NTA agarose column. Gel was stained with Coomassie Brilliant Blue R-250. Arrows: rPcrV protein. (TIF) [file ppat.1012696.s001.tif]

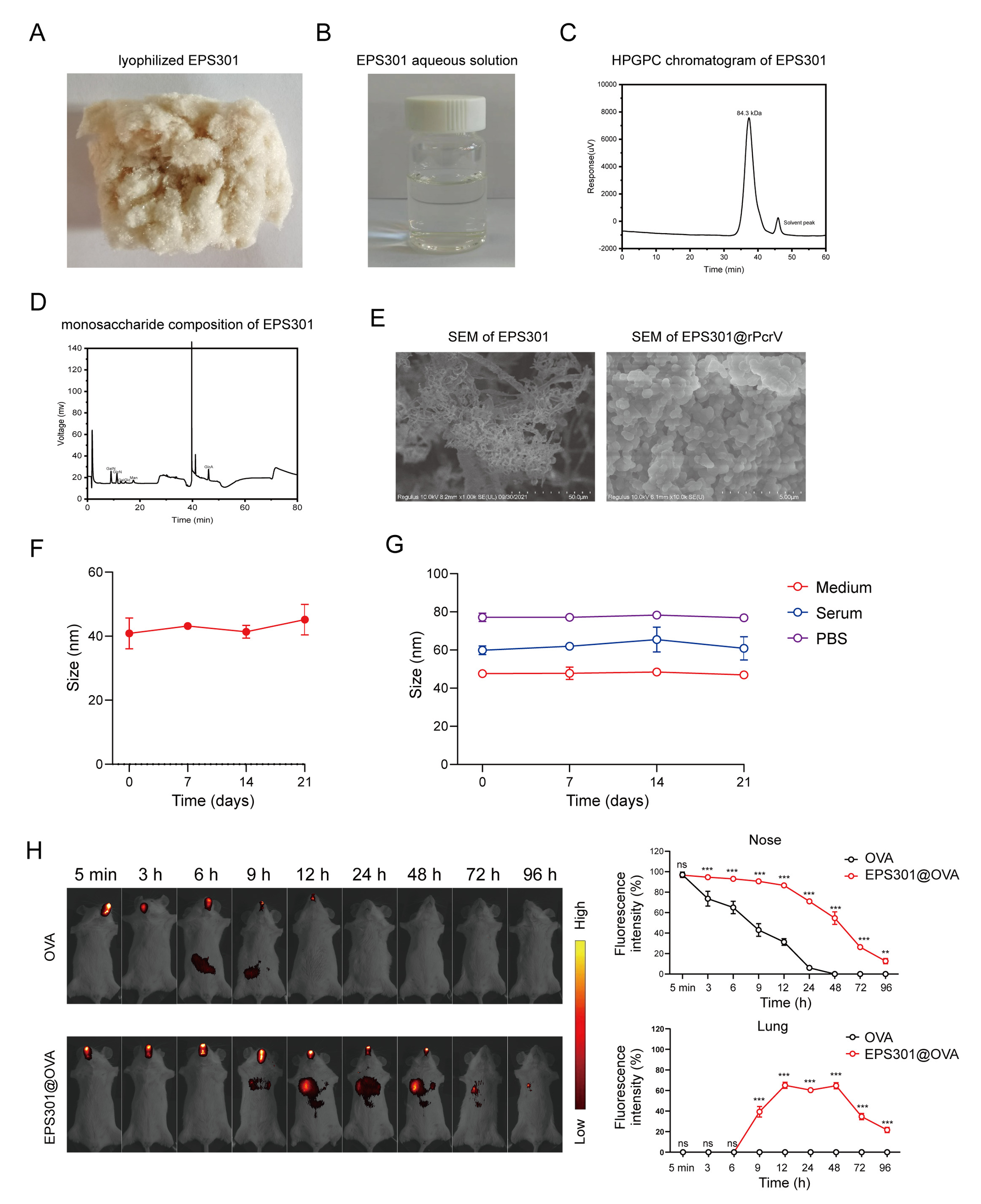

Supplement: S2 Fig — (A) Photo of lyophilized EPS301. (B) Photo of EPS301 aqueous solution. (C) The homogeneity and molecular weight of EPS301. (D) The monosaccharide composition of EPS301. (E) The scanning electron microscopy (SEM) images of EPS301 and EPS301@rPcrV. (F) The solution (1.0 mg/mL) of EPS301 in PBS was prepared, whose particle size, of EPS301 were measured by the dynamic light scattering (DLS) technique using a Nano Brook (Nano Brook, 90 Plus, PALS, U.S). The values presented are the average of three measurements, and the standard deviation was considered as the error range. (G) The solution (1.0 mg/mL) of EPS301 in PBS, serum and 1640 medium were prepared, whose particle size, of EPS301@rPcrV were measured by the dynamic light scattering (DLS) technique using a Nano Brook (Nano Brook, 90 Plus, PALS, U.S). The values presented are the average of three measurements, and the standard deviation was considered as the error range. (H) Representative in vivo fluorescence images of mice at the indicated time points after intranasal administration of free OVA or EPS301@OVA. OVA in both groups labeled with Cy7. Significant differences were calculated with One-way ANOVA followed by Tukey’s multiple comparisons test. ns, not significant, **p < 0.01, ***p < 0.001. Data are presented as means ± SEM. (TIF) [file ppat.1012696.s002.tif]

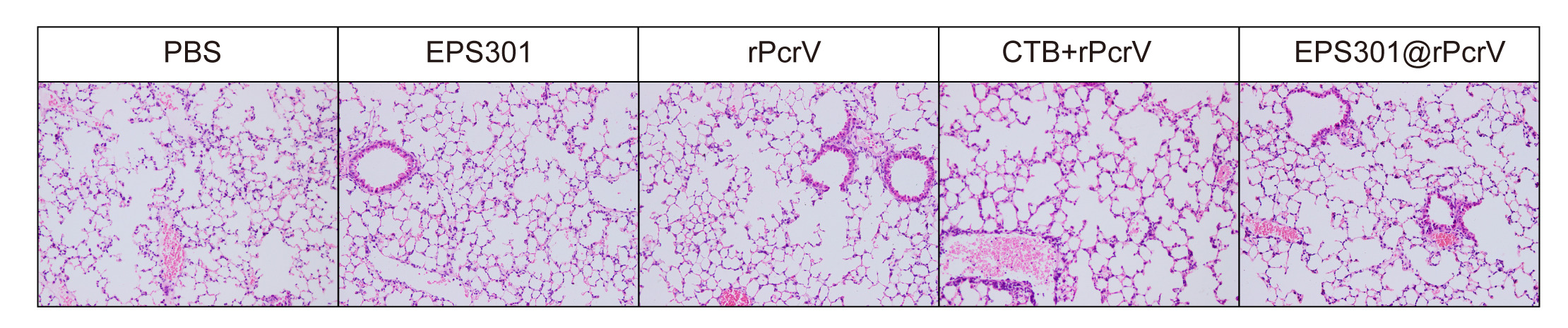

Supplement: S3 Fig — Histological evaluations conducted before challenge. Mice were immunized twice via airway (intranasal, i.n.) administration routes. Animals (3–5 mice per group) were sacrificed on day 7 after boost immunization for histological evaluation of lung sections using light microscopy. Lung specimens were fixed, sectioned, and stained with H&E (n = 3–5). (TIF) [file ppat.1012696.s003.tif]

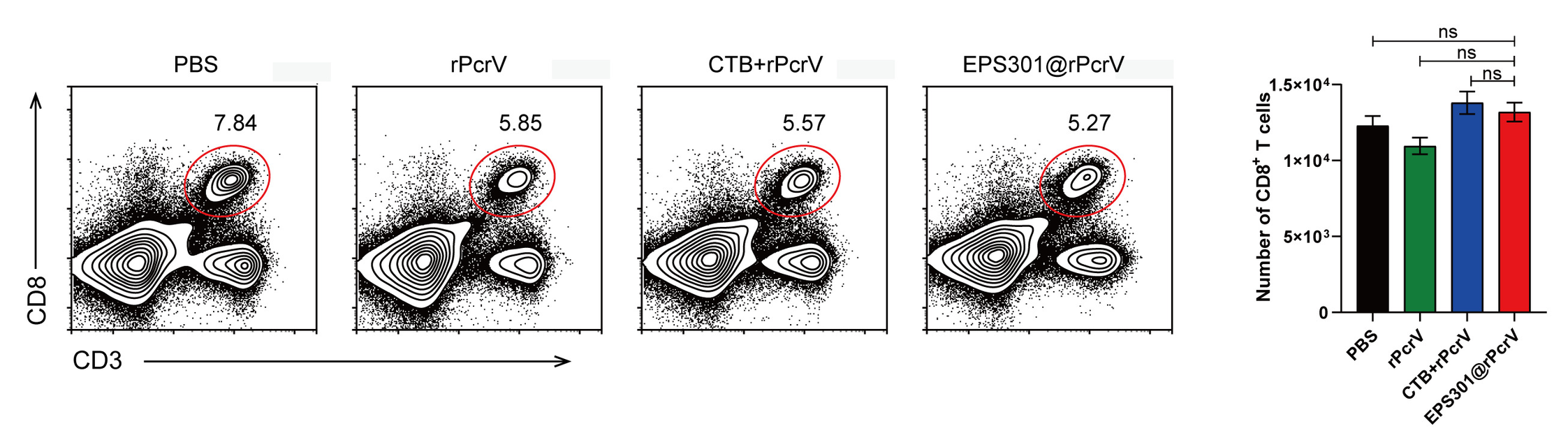

Supplement: S4 Fig — Mice (n = 3-5/group) were immunized (i.n.) twice 14 days apart with rPcrV, CTB+rPcrV or EPS301@rPcrV, with animals receiving PBS served as controls. Vaccinated mice were sacrificed at 12 hours post-challenge on day 7 after the second vaccination lung tissue were prepared. Number of CD8+ T cells were determined by Flow cytometric. Data are presented as means ± SEM. Significant differences were calculated with One-way ANOVA followed by Tukey’s multiple comparisons test. ns, not significant. (TIF) [file ppat.1012696.s004.tif]

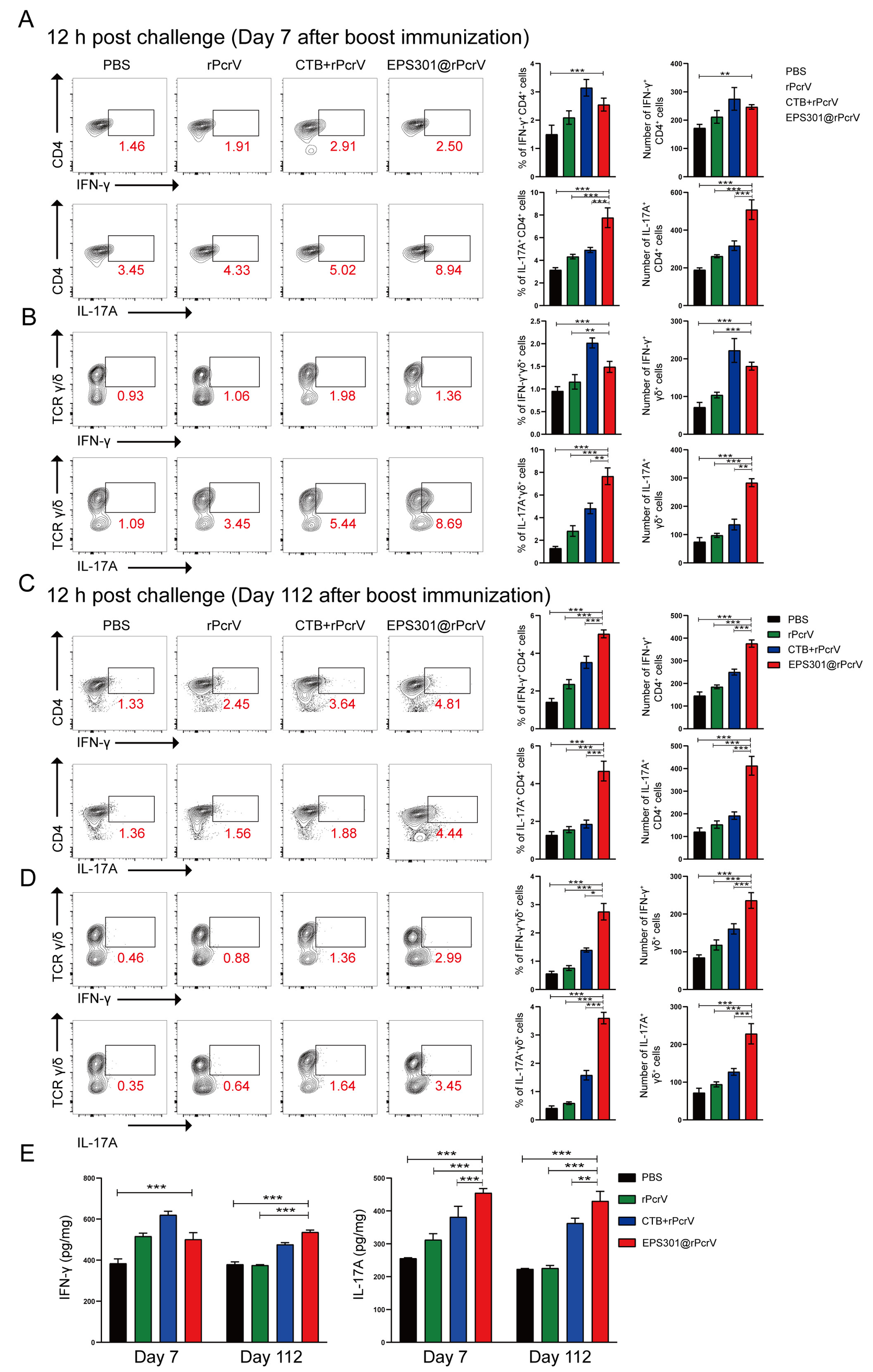

Supplement: S5 Fig — Mice (n = 3-5/group) were immunized (i.n.) twice 14 days apart with rPcrV, CTB+ rPcrV or EPS301@rPcrV, with animals receiving PBS served as controls. Mice were inoculated with 40 μl bacterial slurry (lower dose, 1×10^7^ CFUs of P. aeruginosa PAO1) as above. Vaccinated mice were sacrificed at 12 hours post-challenge on day 7 or day 112 after the second vaccination spleen tissue were prepared. Number of IFN-γ+ CD4+ T cells, IL-17A+ CD4+ T cells (A), IFN-γ+ γδ+ T cells, IL-17A+ γδ+ T cells (B) in lung at 12 hours post-challenge on day 7 after the second vaccination were estimated by intracellular cytokine. Number of IFN-γ+ CD4+ T cells, IL-17A+ CD4+ T cells (C), IFN-γ+ γδ+ T cells, IL-17A+ γδ+ T cells (D) in lung at 12 hours post-challenge on day 112 after the second vaccination were estimated by intracellular cytokine. IFN-γ and IL-17 levels, determined by ELISA in a supernatant of lung tissue homogenate were analyzed. The IFN-γ levels and IL-17A levels (E) in spleen at 12 hours post-challenge on day 7 and day 112 after the second vaccination were determined by ELISA. Data are presented as means ± SEM. Significant differences were calculated with One-way ANOVA followed by Tukey’s multiple comparisons test. *p < 0.05, **p < 0.01, ***p < 0.001. (TIF) [file ppat.1012696.s005.tif]

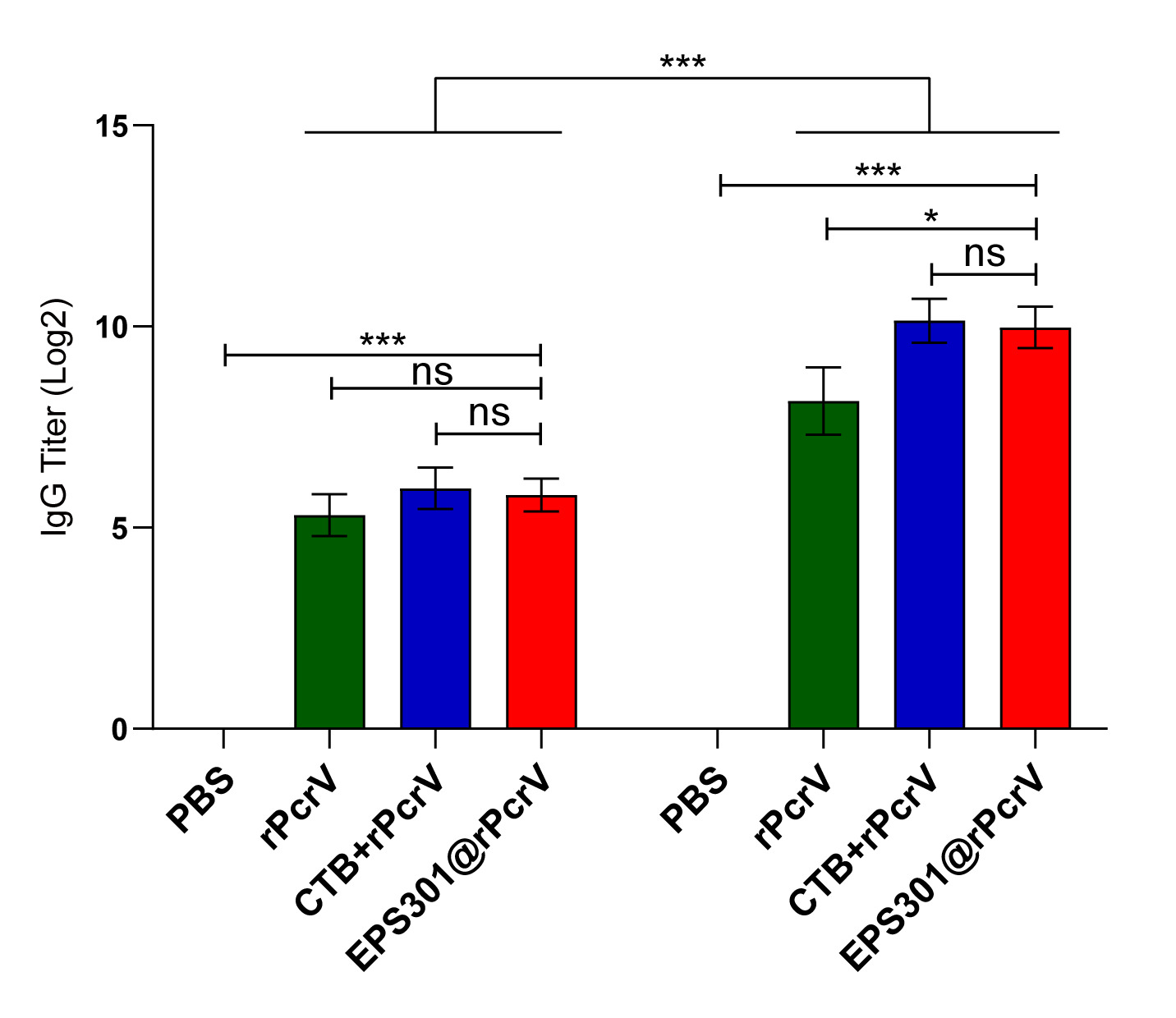

Supplement: S6 Fig — Antigen-specific IgG in serum of the recipient mice and actively immunized mice was detected using ELISA. Data are presented as means ± SEM. Significant differences were calculated with One- or Two-way ANOVA followed by Tukey’s multiple comparisons test. ns, not significant, *p < 0.05, ***p < 0.001. (TIF) [file ppat.1012696.s006.tif]
