# Supplementary material for: Mucosal immunization with the lung Lactobacillus-derived amphiphilic exopolysaccharide adjuvanted recombinant vaccine improved protection against P. aeruginosa infection
Source: PLoS Pathog. 2024 Nov 18;20(11):e1012696. doi: 10.1371/journal.ppat.1012696 (PMC11611261; doi:10.1371/journal.ppat.1012696)
Supplement: Attachment — Submitted filename: Response Letter.docx [file ppat.1012696.s007.docx]

**Response Letter**

Journal: PLOS Pathogens

Manuscript ID: PPATHOGENS-D-24-00238

Manuscript Title: **Mucosal immunization with the lung *Lactobacillus*-****derived amphiphilic exopolysaccharide adjuvanted recombinant vaccine improved protection against *P. aeruginosa* infection**

Author(s): Haochi Zhang^1,2,^^¶^, Shouxin Sheng^1,¶^, Chunhe Li^1^, Xuemei Bao^1^, Lixia Zhao^2^, Jian Chen^1,2^, Pingyuan Guan^2^, Xiaoyan Li^2^, Na Pan^1^, Yanchen Liang^1^, Xueqi Wang^1^, Jingmin Sun^1^, Xiao Wang^1^*

Dear editors

Thank you very much for giving us the opportunity to revise our manuscript. We highly appreciate the constructive comments provided by you and all the reviewers. These comments and suggestions are very helpful. We have addressed all the concerns and issues carefully and have revised the manuscript accordingly. In the revised manuscript, the reviewer’s comments are presented in blue font, and our responses are in black font. All changes made in the revised manuscript are highlighted in yellow. Please let us know if you have any further suggestions.

**Comment 1:** The first major issue raised by all reviewers is the challenge dose/administration reported in the manuscript. The results in Figure 1 and 4 should be more described and discussed.

**Response:** We deeply appreciate the editor and reviewers for highlighting these points. We have added the challenge dose to both the figure legends and the Materials and Methods section. As noted by the reviewer, the IVIS imaging indicated that a significant portion of the vaccine was swallowed or administered down the esophagus, likely due to the large volume administered. To address this, we conducted multiple repetitions of fluorescent in vivo imaging experiments to determine a suitable administration volume. As shown in the revised Fig 1E, a much stronger signal of Cy7-OVA was observed in both the nasal passages and lungs of EPS301@Cy7-OVA inoculated mice compared to those inoculated with Cy7-OVA alone. These results suggest that EPS301 encapsulation effectively penetrates the nasal mucous layer and prolongs antigen retention. We have included these findings in lines 148-151 of the revised manuscript. Additionally, we have added the size of EPS301-formulated *P. aeruginosa* true antigen (rPcrV) in the revised Fig 1D. Furthermore, we thank reviewer #2 for the constructive criticism. In our flow cytometric and ELISA analyses, we initially focused on the trends of IL-17A- or IFN-γ-secreting CD4 T cells and γδ T cells, as well as IL-17A and IFN-γ levels among these four groups, but we overlooked the absolute numbers of these cells. We have repeated these experiments and provided the updated results in revised Fig 4.

**Comment 2:** The second major issue is the difference between the results in Figure 2 and 3. The authors should better explained and discussed these points.

**Response:** Thanks for editor and reviewers pointed this out. It may be our unclear expression caused your confusion. Fig 2 shows the effective protection of EPS301-adjuvanted mice against *P. aeruginosa*. The efficacy of EPS301 as a mucosal adjuvant against *P. aeruginosa* infection was demonstrated by measuring survival rates, bacterial colonization in lungs, bronchoalveolar lavage fluid, and spleen, and lung pathology 7 days after booster immunization. Fig 3 shows that EPS301 is superior to the "gold standard" - CTB in terms of sustained protection, as evidenced by survival rates, bacterial colonization, lung pathology, and serum IgG and lung IgA levels 112 days after booster immunization.

**Comment 3:** The last major issue concerns the infection model. As pointed out by reviewer #3, use of PA01 rather than a T3SS-producing strain should be discussed since lower virulence might impact vaccine efficacy evidencing. In addition, the choice of germline TCR δ^-/-^ mice should be justified.

**Response:** We appreciate the editor and reviewers for pointing this out. PA103 and PA14 have indeed exhibited increased virulence compared to PAO1 in various models. We reviewed numerous articles before choosing the challenge strain. Although less virulent than PA103, PAO1 is also a commonly used strain to model pneumonia[1-2]. Therefore, we chose PAO1 as the challenge strain to study the role of EPS as a mucosal adjuvant in anti-*P. aeruginosa* infection and to investigate the molecular mechanism of EPS301. Based on these results, will further investigate the broad response of this vaccine and the novel adjuvant to other strains of *P. aeruginosa*, such as PA103, PA14, and PA117.

γδ T cells are generally recognized as innate immune cells but actually play key roles in both innate and adaptive immune functions. Additionally, γδ T cells are important in *P. aeruginosa* infection. Therefore, in this study, we used TCR δ^-/-^ mice to investigate the protective mechanism of EPS301. As suggested by the reviewer, we examined the changes in Th17 cells and IgA in WT and TCR δ^-/-^ mice before and after immunization, as well as post-infection. We have included these results in revised Fig 6A and reviewer Fig 3.

Thank you again for your positive comments and valuable suggestions to improve the quality of our manuscript. Please don't hesitate to contact us if you have any questions or comments.

With best wishes.

Yours sincerely.

Xiao Wang

**Response to Reviewer 1’s Comments**

According to the reviewers’ comments, we have made extensive modifications to our manuscript and supplemented extra data to make our results convincing. Thank you again for your positive comments and valuable suggestions to improve the quality of our manuscript.

**Reviewer #1:** The manuscript of Xiao Wang et al reports on nasal immunization against *P aeruginosa* using an encapsulating lactobacillus exopolysaccharide as adjuvant, that was compared to Cholera toxin adjuvant. Ab induction including pulmonary IgA were checked as well as protection efficacy in either direct of adoptive cell transfer challenge tests. Cellular response was assessed by cytokine dosages and flow cytometry analysis of lung and spleen cells populations. Immunization and challenge of IFN, IL17, Gamma delta T cell deficient mice were also performed. Displaying an interesting effect of IL17 deficiency on *P aeruginosa* infection.

Although reporting an interesting work, several points of the manuscript need to be clarified or better presented.

**Major comments:**

**Comment 1**: Figure1 I (imaging) is not supporting comments done in the main text. Firstly, amounts (or signal settings) of fluorescent OVA appear to be different for OVA alone or encapsulated OVA. Secondly, I do not agree with the given interpretation of images (look at 12 hours): although the free OVA is only located in one lung (suggesting a direct bronchial and not nasal administration), adjuvanted OVA is mostly found in the gut or bladder, and also in the liver for a small part. And incidentally not in the spleen throughout the whole experiment. Suggesting rapid clearance and/or degradation of most of the Ag. No data using the true *P aeruginosa* recombinant Ag are provided. That figure should be either completed by another experiment, more extensively commented, or deleted.

**Response**: We appreciate the reviewer pointing this out. As highlighted, the IVIS imaging indicated that a significant portion of the vaccine may have been swallowed or administered down the esophagus, possibly due to the large volume administered. To address this concern, we conducted multiple repetitions of fluorescent in vivo imaging experiments to determine the appropriate administration volume. As depicted in the revised Fig 1E, a notably stronger signal of Cy7-OVA was observed in the lungs of mice inoculated with EPS301@Cy7-OVA compared to those inoculated with Cy7-OVA alone. These findings suggest that EPS301 encapsulation enables effective penetration of the nasal mucous layer and prolongs antigen retention. We have included these descriptions in lines 148-151 of the revised manuscript. Additionally, we have provided some characteristics of the true *P. aeruginosa* recombinant antigen (rPcrV) formulated with EPS301 in the revised Fig 1D.

**Comment 2**: I'm unable to understand the difference between survival curves in Fig 2 and Fig 3. Please clarify.

**Response**: Thanks for reviewers pointed this out. It may be our unclear expression caused your confusion. Fig 2 shows the effective protection of EPS301-adjuvanted mice against *P. aeruginosa*. The efficacy of EPS301 as a mucosal adjuvant against *P. aeruginosa* infection was demonstrated by measuring survival rates, bacterial colonization in lungs, bronchoalveolar lavage fluid, and spleen, and lung pathology 7 days after booster immunization. Fig 3 shows that EPS301 is superior to the "gold standard", CTB, in terms of sustained protection, as evidenced by survival rates, bacterial colonization, lung pathology, and serum IgG and lung IgA levels 112 days after booster immunization.

**Minor comments:**

**Comment 1**: Size of nano-particles of EPS301 encapsulating protein is far bigger than that of EPS301 alone but data are from BSA and not from the true Ag used and a single ratio of EPS versus protein seems to have been used (lines574-579). It would be of interest to look at the change of particle size according to various ratios or to the constant size due to mechanical self-assembly constraints.

**Response**: As the reviewer suggested, we have now included the sizes of the true antigen formulated with EPS301 in the revised Fig 1. Additionally, we conducted measurements of particle sizes at various ratios (Reviewer Fig 1). Dynamic light scattering (DLS) results revealed that the size of self-assembled EPS301 was approximately 24 ± 6 nm, EPS301@OVA was about 41.08 ± 5 nm, and EPS301@rPcrV was around 43.10 ± 5 nm at a ratio of 1 mg/ml. At a ratio of 0.5 mg/ml, the size of self-assembled EPS301 was approximately 24.4 ± 6 nm, EPS301@OVA was about 37.86 ± 5 nm, and EPS301@rPcrV was approximately 40.21 ± 5 nm. Similarly, at a ratio of 0.25 mg/ml, the size of self-assembled EPS301 was approximately 20.24 ± 6 nm, EPS301@OVA was about 37.06 ± 5 nm, and EPS301@rPcrV was around 38.57 ± 5 nm. Importantly, there was no significant difference observed among these ratios. Therefore, we only shown the particle size of 1mg/ml in manuscript.

**Revised Fig 1.** Sizes of EPS301, EPS301@OVA and EPS301@rPcrV nanoparticles.

**Comment 2**: It would be mandatory to give details of Ag concentration/ mouse and Ag/adjuvant ratio as well (and not those of BSA as the development model).

**Response**: We thank the reviewer for the constructive criticism. We have provided details regarding the antigen concentration per mouse and the antigen/adjuvant ratio in lines 624-625 of the revised manuscript.

**Comment 3**: Lung pathological findings of challenged mice are provided, but not from immunized mice before any challenge. that would also be of interest.

**Response**: The reviewer has offered a constructive suggestion. As depicted in reviewer Fig 2, histological evaluations before challenge were included. No significant inflammation was observed among these groups.

**Reviewer Fig 2.** Illustrates histological evaluations conducted before challenge. Mice were immunized twice via airway (intranasal, i.n.) administration routes. Animals (3-5 mice per group) were sacrificed on day 7 after boost immunization for histological evaluation of lung sections using light microscopy. Lung specimens were fixed, sectioned, and stained with H&E (n=3-5).

**Comment 4**: Fig 3 F (12h challenge) deserves more comments as supporting efficacy of the proposed vaccine: if pictures are representative, Ag alone is poorly protective whereas EPS301@rPcrV is totally protective. Cholera toxin exhibits a global inflammation. As mentioned above, pathological samples without challenge would have allowed to decipher between something due to Cholera toxin itself or a different answer to *P. aeruginosa* challenge after use of CTB.

**Response**: We appreciate the constructive criticism from the reviewer. As demonstrated by the pathological samples before challenge (Reviewer Fig 2), there was no evidence of inflammation in CTB-adjuvanted vaccinated mice prior to challenge. However, we observed that Cholera toxin induces global inflammation post-challenge, particularly evident 112 days after the second immunization. Considering the lower survival rate, higher bacterial loading, and widespread inflammation observed in the long-term protection conferred by CTB, it indicates that CTB is not effective in providing sustainable protection.

**Response to Reviewer 2’s Comments**

We thank the reviewer for your valuable comments and indications. We have amended and strengthened our manuscript thanks to these valuable remarks. We have responded to the reviewers’ comments on a point-by-point basis. Thank you again for your positive comments and valuable suggestions to improve the quality of our manuscript.

**Reviewer #2:** The authors evaluated protection efficacy and mechanism of a vaccine composed of PcrV and Lactobacillus derived exopolysaccharide as an adjuvant (EPS301@rPcrV). The vaccine conferred protection against *P. aeruginosa* lung infection. The authors further demonstrated that the vaccine elicited antibody against PcrV. By adaptive transfer and using IL-17A knockout mice, they demonstrated that CD4 and γδ T cells mediated IL-17 production play important roles in the protection.

**Major comments:**

**Comment 1**: Fig. 1I: At 12 hours post the inoculation, the Cy7 signal in the lung is stronger in the mouse received Cy7-OVA than that received EPS301@Cy7-OVA. It seems Cy7-OVA retain longer in the lung, which should be discussed. Meanwhile, which organs are the Cy7-OVA distributed after 12 hours? It does not seem to be in the spleen.

**Response**: We thank the reviewer for the constructive criticism. In response to the concern raised regarding IVIS imaging showing a significant portion of the vaccine being swallowed or administered down the esophagus, possibly due to the large volume administered, we conducted repeated experiments of fluorescent in vivo imaging to determine the appropriate administration volume. As depicted in the revised Figure 1E, a much stronger signal of Cy7-OVA was observed in the lungs of mice inoculated with EPS301@Cy7-OVA compared to those inoculated with Cy7-OVA alone. These findings suggest that EPS301 encapsulation enables effective penetration of the nasal mucous layer and prolongs antigen retention. We have incorporated these descriptions into lines 148-151 of the revised manuscript.

**Comment 2**: Fig. 4: What was the challenge dose?

Fig. 4A, B: The IFN-γ+ CD4+ T cells in all the samples in the day 112 group were higher than the corresponding ones in the day 7 group, however, the IL-17A+ CD4+ T cells were lower. Why the IFN-γ response was stronger after longer period of time after vaccination? These results need to be confirmed and explained.

The same question for the Fig. 4C, D: the IFN-γ+ γδ+ T cells and IL-17A+ γδ+ T cells were higher in the day 112 groups than those in the day 7 groups.

The overall IFN-γ level was lower in the CTB+ rPcrV immunized mice in the day 112 groups than that in the day 7 group (Fig. 4E), and the IFN-γ level was similar in the EPS301@rPcrV immunized mice in the day 112 groups than that in the day 7 group. These results are contradictory to the higher numbers and percentages of IFN-γ+ cells in the day 112 groups (Fig. 4A, 4C).

**Response**: We appreciate the constructive criticism from the reviewer, and we apologize for the errors in our flow cytometric and ELISA analyses. To address this, we have included the challenge dose in both the figure legends (Lines 271-273) and the Materials and Methods section (Lines 687-689). Previously, our focus was primarily on the trends of IL-17A- or IFN-γ-secreting CD4 T cells or γδ T cells, as well as IL-17A and IFN-γ among these four groups, without considering the absolute number of these cells. In response to this feedback, we repeated these experiments and have provided the updated results in the revised Fig 4. Additionally, we have analyzed the lung TRM cells response induced by EPS301, which is illustrated in Fig 5J.

**Comment 3**: Fig. 5A: When were the sera collected (how many days post the second immunization)? Based on the method description, after incubation with PAO1, the extracellular bacteria were washed away and the macrophage cells were lysed, followed by determination of live intracellular bacterial numbers by plating. Thus the experiment was the opsonophagocytosis assay, not opsonophagocytosis killing activity as stated in the figure legend.

If this was the opsonophagocytosis killing assay, it is strange that the bacterial numbers remained similar after dilution of the sera from immunized mice when 10^4^ CFU of PAO1 was used.

**Response**: We appreciate the constructive criticism from the reviewer and apologize for the mistakes in the description. It is indeed the opsonophagocytosis assay, not opsonophagocytosis killing activity. We have corrected this description in lines 292-298 of the revised manuscript.

**Comment 4**: Fig. 5C. A previous report demonstrated that anti-PcrV IgG was able to confer protection in mice (Nat Med. 1999. 5(4):392-8. doi: 10.1038/7391). An explanation should be provided.

**Response**: Thanks for reviewer’s question. We thoroughly examined the reference provided by the reviewer. The discrepancies in these results may arise from differences in vaccine type, the P. aeruginosa challenge strain, or the mouse strain used across the studies. Our opsonophagocytosis assay suggests that transferring larger quantities of anti-rPcrV serum from immunized mice might offer partial protection. Further investigation is necessary to elucidate the specific roles of B and T cells induced by EPS301@rPcrV immunization in mediating protection. Additionally, we have included these explanations in lines 512-518 of the revised manuscript.

**Comment 5**: For the T cell transfer assays, why were the numbers of γδ T cells (10000) and CD4+ T cells (50000) different?

**Response**: Thanks for reviewer’s question. There are two reasons for this decision: Firstly, we conducted experiments using different numbers of γδ T cells and CD4 T cells for transfer and found that 10,000 γδ T cells or 50,000 CD4 T cells administered intravenously were able to exert protective effects. Secondly, CD4 T cells typically constitute over 90% of the total T cell population, whereas γδ T cells represent a smaller proportion. Therefore, we ultimately decided to use 10,000 γδ T cells or 50,000 CD4 T cells for adoptive transfer.

**Comment 6**: Transfer of CD4+ T cells resulted in approximately 10-fold less bacteria in the lungs than those received γδ T cells (Fig. 5H and 6F), however, the IL-17A levels was similar between the two groups (Fig. 5I and 6G). Does it mean that besides IL-17 the CD4+ T cells provided other mechanisms of protection?

**Response**: Thanks for reviewer’s question. Indeed, various lymphoid cell types have been identified whose functions can overlap with those of conventional T cells, including γδ T cells, ILC cells, NKT cells, and other immune cells. In this study, we focused on detecting the Th1, Th17, and γδ T cell responses induced by EPS301. However, besides Th17 cells, it is possible that other immune responses induced by EPS301 also contribute to protection. In our future studies, we plan to delve deeper into the protective mechanism induced by EPS301 to comprehensively understand its immunological effects.

**Minor comments:**

**Comment 1**: Fig. 2D. The scale bar is missing.

**Response**: We apologize for the mistakes. We have added scale bar in revised Fig 2D.

**Comment 2**: Line 426 No need to use the full name “*Pseudomonas aeruginosa*” here.

**Response**: Thanks for reviewer’s suggestion. We have deleted the full name “*Pseudomonas aeruginosa*” in line 445.

**Response to Reviewer 3’s Comments**

We thank the reviewer for your valuable comments and indications. According to the reviewers’ comments, we have made extensive modifications to our manuscript and supplemented extra data to make our results convincing. Thank you again for your positive comments and valuable suggestions to improve the quality of our manuscript.

**Reviewer #3:** The paper by Zhang et al report on a Lactobacillus eps vaccine that elicits IgA as well as lung Th17 and γδ 17+ cells. This vaccine provides protection against a PA01 challenge. The data are interesting but preliminary. Moreover, the cellular immunology is not state of the art.

**Major comments:**

**Comment 1**: The IVIS imaging shows a lot of the vaccine is swallowed or administered down the esophagus. This is likely due to the large volume administered. Was the 40 uL delivered in a single nare or split across left and right nare? Volumes above 15 ul are more likely to aspirated into the lung/stomach. Also what anesthesia was used?

**Response**: Thanks for reviewer’s suggestion. We repeated the experiments of fluorescent in vivo imaging multiple times and determined that the suitable volume of administration is 20 μl per mouse. As shown in the revised Figure 1E, a much stronger signal of Cy7-OVA was observed in the lungs of mice inoculated with EPS301@Cy7-OVA compared to those inoculated with Cy7-OVA alone. These results suggest that EPS301 encapsulation effectively penetrates the nasal mucous layer and prolongs antigen retention. We have added these descriptions to lines 148-151 of the revised manuscript. Additionally, we specified that mice were anesthetized with isoflurane, and this information has been added to lines 605-606 of the revised manuscript.

**Comment 2**: The authors use a PA01 challenge strain. It is my understanding this strain does not efficiently express the Type III secretion system and some labs have this used PA103 or PA14 to model ventilator associated pneumonia which is associated with Type III secretion.

**Response**: We appreciate the reviewers for pointing this out. PA103 and PA14 do exhibit increased virulence compared to PAO1 in various models. We reviewed numerous articles before choosing the challenge strain. Although less virulent than PA103, PAO1 is a strain commonly used to model pneumonia[1-2]. Therefore, we chose PAO1 as the challenge strain to study whether EPS301, as a mucosal adjuvant, plays an important role in anti-*P. aeruginosa* infection and to investigate the molecular mechanism of EPS301. Additionally, based on these results, we will next investigate the broad response of this vaccine and the novel adjuvant to other strains of *P. aeruginosa*, such as PA103, PA14, and PA117.

**Comment 3**: It unclear if the authors are eliciting lung TRM cells. This would need to be defined by exclusion of anti-CD45 IV staining. Additionally, TRM cells are CD69+, CXCR6+, S1PR1 lo, KLF2 lo. If these cells are elicited – do they contract over time?

**Response**: This is a very good suggestion. The results of adoptive transfer demonstrated that transferring pulmonary CD4^+^ or γδ^+^ T cells alone provided protection against *P. aeruginosa* infection. We hypothesized that EPS301-adjuvanted vaccination might elicit a lung TRM cell immune response. Strikingly, immunized lung CD4^+^ or γδ^+^ T cells expressed CD44 and CD69, which are considered markers for lung TRM cells[3-4]. We have included these results in the revised Fig 5. Additionally, in future studies, we will investigate other molecules expressed by TRM cells, such as Klf2, S1pr1, and Cxcr6.

**Comment 4**: Germline TCR δ^-/-^ mice have known developmental differences in alpha-beta cells. The authors would need to show that Th17 cells and IgA are still present in these mice.

**Response**: Thanks for reviewer’s suggestion. As shown in the revised Fig 6A, there was no significant difference in the number and percentage of Th17 cells between TCR δ-deficient and WT mice before immunization. However, the number and percentage of Th17 cells were much higher in WT mice than in TCR δ-deficient mice both after immunization and post-infection. Additionally, we observed similar changes in antigen-specific IgA levels in the lungs before and after immunization, as illustrated in Reviewer Fig 3. This proves to some extent that γδ T cell is the bridge between innate immune response and adaptive immune response.

Reviewer Fig 3. rPcrV specific IgA before and post immunization in lung of TCR δ^-/-^ mice and WT mice.

References

1.	Jiang M, Yao J, Feng G. Protective effect of DNA vaccine encoding pseudomonas exotoxin A and PcrV against acute pulmonary *P. aeruginosa* Infection. PLoS One. 2014; 9(5):e96609 <https://doi.org/10.1371/journal.pone.0096609> PMID: PMC4006881

2.	Li P, Wang X, Sun X, Guan Z, Sun W. Outer Membrane Vesicles Displaying a Heterologous PcrV-HitA Fusion Antigen Promote Protection against Pulmonary *Pseudomonas aeruginosa* Infection. mSphere. 2021; 6(5):e0069921 <https://doi.org/10.1128/mSphere.00699-21> PMID: PMC8510544

3.	Iwanaga N, Chen K, Yang H, Lu S, Hoffmann JP, Wanek A, et al. Vaccine-driven lung TRM cells provide immunity against Klebsiella via fibroblast IL-17R signaling. Sci Immunol. 2021; 6(63):eabf1198 <https://doi.org/10.1126/sciimmunol.abf1198> PMID: PMC8796208

4.	Amezcua Vesely MC, Pallis P, Bielecki P, Low JS, Zhao J, Harman CCD, et al. Effector T(H)17 Cells Give Rise to Long-Lived T(RM) Cells that Are Essential for an Immediate Response against Bacterial Infection. Cell. 2019; 178(5):1176-88.e15 <https://doi.org/10.1016/j.cell.2019.07.032> PMID: PMC7057720
