# Supplementary material for: Mucosal immunization with the lung Lactobacillus-derived amphiphilic exopolysaccharide adjuvanted recombinant vaccine improved protection against P. aeruginosa infection
Source: PLoS Pathog. 2024 Nov 18;20(11):e1012696. doi: 10.1371/journal.ppat.1012696 (PMC11611261; doi:10.1371/journal.ppat.1012696)
Supplement: Attachment — Submitted filename: Response Letter.docx [file ppat.1012696.s008.docx]

**Response Letter**

Journal: PLOS Pathogens

Manuscript ID: PPATHOGENS-D-24-00238

Manuscript Title: **Mucosal immunization with the lung *Lactobacillus*-****derived amphiphilic exopolysaccharide adjuvanted recombinant vaccine improved protection against *P. aeruginosa* infection**

Author(s): Haochi Zhang^1,2,^^¶^, Shouxin Sheng^1,¶^, Chunhe Li^1^, Xuemei Bao^1^, Lixia Zhao^2^, Jian Chen^1,2^, Pingyuan Guan^2^, Xiaoyan Li^2^, Na Pan^1^, Yanchen Liang^1^, Xueqi Wang^1^, Jingmin Sun^1^, Xiao Wang^1^*

Dear editor

Thank you very much for giving us the opportunity to revise our manuscript. We highly appreciate the constructive comments provided by you and the reviewers. In response to the concerns raised by the editor and Reviewer 1 regarding fluorescence imaging, we conducted a new analysis using the true antigen from *P. aeruginosa*, rPcrV, and have presented these results in Fig 1 of the revised manuscript. Based on Reviewer 3’s suggestions for the statistical analysis section, we have refined the statistical analysis accordingly. Additionally, we have addressed all other comments raised by the reviewers individually. All changes made in the revised manuscript are highlighted in yellow.

Thank you again for your positive comments and valuable suggestions to improve the quality of our manuscript.

With best wishes.

Yours sincerely.

Xiao Wang

**Response to Reviewer 1’s Comments**

According to the reviewers’ comments, we have made extensive modifications to our manuscript and supplemented extra data to make our results convincing. Thank you again for your positive comments and valuable suggestions to improve the quality of our manuscript.

**Reviewer #1:** Several comments have been addressed, leading to changes improving the manuscript. But some points remain difficult to understand and others could be further improved.

**Major comments:**

**Comment 1**: Dealing with Figure 1. The response refers to the text line 148-151 as further explanation given. "In addition, EPS301 encapsulation could effectively penetrate the nasal mucous layer and prolonged antigen retention " That only sentence can hardly make clear the new data from the Figure 1 E. On that figure announced from new experiments, no signal is observed with OVA, whereas the signal from EPS301@ova in the lung was detected from T 12 hours and negative before. On the left part (images), whole mouse body imaging should be given, at least for the most significant time points, instead of cropped images. To be sure that signal is from the lungs and not liver or spleen and to illustrate the relative amount ending in the guts.

**Response**: We appreciate the constructive comments from the reviewer and apologize for the inaccuracies in our previous description and the incomplete representation of the images. We have re-evaluated this portion of the experiment using the true antigen from *P. aeruginosa*, rPcrV, and updated our results accordingly. As shown in revised Fig 1, in the group of mice immunized with rPcrV alone, nasal cavity fluorescence almost completely disappeared within 12 hours after intranasal administration. In contrast, in the EPS301@rPcrV immunization group, fluorescence was still detectable in the nasal cavity at 96 hours post-immunization. Additionally, the fluorescence signal intensity in the lungs of the EPS301@rPcrV immunization group was significantly higher than that of the group immunized with rPcrV alone. Furthermore, we dissected mice at different time points to assess fluorescence in major organs. The results showed that the EPS301@rPcrV group had significantly higher fluorescence in both the nasal cavity and lungs compared to the rPcrV-only group. Fluorescence in other organs was predominantly observed in the liver, likely due to liver metabolism. To further demonstrate the general applicability of EPS301 in prolonging antigen release, we labeled OVA with Cy7 and compared the retention times of the antigen alone versus those formulated with EPS301 following intranasal administration. As shown in the S3 Fig H, the results confirmed that EPS301-based nanoparticles significantly extended the antigen release. In summary, EPS301 encapsulation could effectively enhance antigen adhesion to the nasal and pulmonary mucosal tissues, thereby prolonging antigen retention.

**Comment 2**: Concerning the difference between survival curves in fig 2 and 3. Explanations are given in the answer to the comment but not in the manuscript. Moving that point as a minor issue now. BTW, are the 2 missing curves on fig 2B totally negatives or not done?

**Response**: Thanks for the reviewer's suggestions. We have added these explanations in lines 187-190, 224-226, and 553-557 of the revised manuscript.

In Fig 2B, mice in the PBS and EPS301 groups (n=10) died totally within 32 hours after *P. aeruginosa* infection.

**Minor comments:**

**Comment 1**: Concerning the size of the particles. Additional information has been provided and the choice of the 1mg/ml concentration for particle synthesis justified. If the I. N. administration was done at twice that concentration, the description of immunogen preparation should mention it and how the concentration was done.

**Response**: Thanks for reviewers pointed this out. We have provided a more accurate and detailed description of the preparation process for EPS301@rPcrV nanoparticles in lines 651-660 of the revised manuscript. A stock solution of EPS301@rPcrV was prepared at a concentration of 10 mg/ml. If a higher dosage is required for intranasal immunization in mice, the concentration can be diluted accordingly. The immunization doses and the method for preparing EPS301@rPcrV nanoparticles have now been added to lines 650-659 and 682-687 of the revised manuscript.

**Comment 2**: Concerning amount of Ag, adjuvant and ratio. The sentence added in the manuscript does not answer the comment: ""intranasal (i.n.) route (volume 40 μl, antigen, 2mg/kg, and EPS301, 2mg/kg)"" I can't believe that doses have been calculated from the weight of each mouse. If kg is a mistyping for ml, please correct.

**Response**: Thanks for reviewers pointed this out. We have provided details regarding the antigen concentration per mouse and the antigen/adjuvant ratio in lines 685-687 of the revised manuscript.

**Comment 3**: Histology of lungs before any challenge. New data provided demonstrate minimal inflammation in the EPS301@rPcrV group or no inflammation in the other. That should be kept in line with the inflammation obtained post challenge from CTB (see comment4 and answer) and deserves in the manuscript not only a comment but some discussion on the mechanism involved.

**Response**: Thank you for the reviewer’s suggestions. We repeated the H&E staining experiment before challenge to further evaluate whether there was inflammation in the lungs of mice after immunization. The H&E results indicated no significant inflammation in the lungs of any group before *P. aeruginosa* challenge. We speculate that the minimal inflammation observed previously in the EPS301@rPcrV group might be due to individual differences among the mice. We added these results to S3 Fig and provided an explanation in lines 190-192 of the revised manuscript.

Following your suggestion, we have elaborated on the potential causes of inflammation in the CTB immunization group after *P. aeruginosa* infection in the Discussion section of the revised manuscript (lines 511-515).

L138-141 OK minor Fig 1B left TEM of EPS301 If available, a snapshot with a better focused beam should be better

**Response**: The clearer images were provided in Fig 1 A.

L153-191 OK but some concerns on the figure E

**Response**: We re-evaluated and described these results in lines 148-170 of the revised manuscript.

L348-354 OK but nature and rationale for FTY720 (fingolimod) use is not given in the manuscript. Amount used is also not given. All readers are not familiar with selective immunosuppression.

**Response**: The rationale for FTY720 (fingolimod) was provided in lines 356-357 of the revised manuscript. Additionally, we added the FTY720-treatment in Materials and Methods (Lines 774-778).

L442 OK A mention of the severity of ocular infection with *P aeruginosa* could be added.

**Response**: We added the mention and reference of the severity of ocular infection with *P aeruginosa* in lines 493-494 of the revised manuscript.

L531-536 OK but the mention of "several papers" lead to add at least the reference of a review.

**Response**: We apologize for our mistakes. We added relevant references in lines 591-593 of the revised manuscript.

L539-542 OK "permanent" could be added before elevated levels of IL17.

**Response**: We added the “permanent” before elevated levels of IL-17A in line 598 of the revised manuscript.

L656-657 OK btw L658, expression of FCS as 5% final is more usual.

**Response**: We re-described this section in lines 720-721 of the revised manuscript.

L693-895 OK the rationale for the use of anti Fc gamma receptors might be given.

**Response**: We added the rationale for the use of anti Fc gamma receptors in lines 758-759 of the revised manuscript.

**Response to Reviewer 3’s Comments**

We thank the reviewer for your valuable comments and indications. We have amended and strengthened our manuscript thanks to these valuable remarks. Thank you again for your positive comments and valuable suggestions to improve the quality of our manuscript.

**Reviewer #2:** The authors have addressed by concerns.

**Major comments:**

**Comment**: In the statistical analysis section, the authors should specify why they used ANOVA vs Kruskal-Wallis. Also which multiple comparisons test was used such as Dunn's, Hol-Sidak etc. If different tests were used with specific Figures- this information should be in the Figure Legend.

**Response**: We thank the reviewer for the constructive comments. We rewrite the section of Statistical analysis. GraphPad software program was used for data processing and analysis. P values < 0.05 were considered statistically significant. Comparisons between two normally distributed groups were performed by simple two-tailed unpaired Student’s t test, and other analyses for unequal variance were determined by Mann-Whitney U test. For multiple groups comparisons, we used One- or Two- way analysis of variance (ANOVA) with Tukey’s post hoc analysis for equally distributed groups. The data for survival test were analyzed by Wilcoxon log-rank survival test. Values are represented as means ± SEM. P values are annotated as follows: *p < 0.05, **p < 0.01, ***p < 0.001. Additionally, we added the statistical analysis in Figure legend.
