# Supplementary material for: Mucosal immunization with the lung Lactobacillus-derived amphiphilic exopolysaccharide adjuvanted recombinant vaccine improved protection against P. aeruginosa infection
Source: PLoS Pathog. 2024 Nov 18;20(11):e1012696. doi: 10.1371/journal.ppat.1012696 (PMC11611261; doi:10.1371/journal.ppat.1012696)
Supplement: Attachment — Submitted filename: Response Letter.docx [file ppat.1012696.s009.docx]

**Response Letter**

Journal: PLOS Pathogens

Manuscript ID: PPATHOGENS-D-24-00238R2

Manuscript Title: **Mucosal immunization with the lung *Lactobacillus*-****derived amphiphilic exopolysaccharide adjuvanted recombinant vaccine improved protection against *P. aeruginosa* infection**

Author(s): Haochi Zhang^1,2,^^¶^, Shouxin Sheng^1,¶^, Chunhe Li^1^, Xuemei Bao^1^, Lixia Zhao^2^, Jian Chen^1,2^, Pingyuan Guan^2^, Xiaoyan Li^2^, Na Pan^1^, Yanchen Liang^1^, Xueqi Wang^1^, Jingmin Sun^1^, Xiao Wang^1^*

Dear editor

We are very grateful to you for giving us an opportunity to revise our manuscript. We appreciate you very much for your positive and constructive comments and suggestions on our manuscript entitled “Mucosal immunization with the lung *Lactobacillus*-derived amphiphilic exopolysaccharide adjuvanted recombinant vaccine improved protection against *P. aeruginosa* infection” (ID: PPATHOGENS-D-24-00238R2). We have studied reviewers’ comments carefully and tried our best to revise our manuscript (marked in yellow) according to the comments. Please contact me if there are any questions.

With best wishes.

Yours sincerely.

Xiao Wang

**Response to Reviewer 1’s Comments**

We thank the reviewer for your valuable comments and indications. We have amended and strengthened our manuscript thanks to these valuable remarks. Thank you again for your positive comments and valuable suggestions to improve the quality of our manuscript.

**Reviewer #1:** Authors performed new experiments to comply with referee's queries, displaying new interesting features. Details have been also corrected like quality of some images. The blocking point of the fig 1 is now solved.

The manuscript is now acceptable for publication.

**Major comments:**

I would only ask for a minor modification. New in vivo imaging clearly show an unexpected kinetics following nose administration of the encapsulated immunogen. The same time there is some better nose retention than using Ag alone, a secondary move of the NIR signal to the lung after one day is observed. No comments on the potential mechanism of that phenomenon is done. I would propose two hypothesis to discuss: a) a drying of the product instillated in the nose with a subsequent inhalation of crusts when breathing. a) a nose obstruction due to a local reaction, leading to some snoozing with inhalation at the inspiratoty phase of coughing.

The difference in protection according to the time is now better depicted and correctly linked to the problem of reaching an enduring immune response, rightly considered to be a major axis for future works.

**Response**: Thank you for the reviewer’s suggestion. Fluorescent imaging analysis revealed that the EPS301-encapsulated immunogen can prolong antigen release. Six hours after intranasal immunization, the fluorescent signal was observed to undergo secondary move to the lungs. We consider the reviewer’s hypothesis that 'the drying of the instilled EPS301@rPcrV in the nose, followed by the inhalation of crusts during breathing, leading to the secondary transfer of the fluorescent signal', may be one of the potential reasons. It is also possible that the EPS301-encapsulated immunogen promotes antigen accumulation in the lungs, which suggests that EPS301 could function as a mucosal adjuvant specifically targeting the lungs. Nonetheless, the precise mechanism remains unclear, and we will further explore the specific mechanism of EPS301 in future research. The discussion of a secondary move to NIR signal to the lung was added in the Discussion section of the revised manuscript (lines 544-551). Once again, we express our gratitude to the reviewer for this constructive suggestion.
